# Effect of Spectacle Lenses with Highly Aspherical Lenslets on Binocular Vision and Accommodation in Myopic Children with and without Intermittent Exotropia

**DOI:** 10.1155/2022/9306848

**Published:** 2022-10-12

**Authors:** Zhou Fengchao, Li Xue, Huang Yingying, Li Yuhao, Zhang Jiali, Chen Hao, Bao Jinhua

**Affiliations:** ^1^Eye Hospital and School of Ophthalmology and Optometry, Wenzhou Medical University, Wenzhou, Zhejiang, China; ^2^National Clinical Research Center for Ocular Diseases, Wenzhou, Zhejiang, China

## Abstract

**Purpose:**

To evaluate the influence of spectacle lenses with highly aspherical lenslets (HAL) on binocular vision and accommodation in myopic children with intermittent exotropia (IXT) and compare the changes after wearing HAL in binocular vision and accommodation in myopic children with or without IXT.

**Method:**

Forty myopic subjects aged 8–12 years were recruited: 20 with IXT and 20 visually normal children. Stereoacuity, phoria, accommodative facility, fusional vergence, vergence facility, near point of convergence, amplitude of accommodation, and accommodative response (AR) were measured by wearing HAL or single vision spectacle lenses (SVL) in a random order after adapting for 20 minutes. Accommodative microfluctuation (AMF) was defined as the standard deviation of AR. Changes in binocular vision and accommodation after wearing HAL were compared between the two groups.

**Results:**

No significant differences were found in binocular vision after wearing HAL versus SVL in either group (all *P* > 0.05). A greater AMF was found after wearing HAL than after wearing SVL in both groups (0.04 D, 95% confidence interval (CI), 0.03 to 0.05 D, *P* < 0.001 for the IXT group; 0.05 D, 95% CI, 0.03 to 0.07 D, *P* < 0.001 for the visually normal group); however, the other accommodation parameters did not change significantly (all *P* > 0.05). There were no differences in the changes after wearing HAL in any parameter between the two groups (all *P* > 0.05).

**Conclusion:**

HAL did not significantly change the binocular vision and accommodation for myopic children with or without IXT except for AMF in the short term.

## 1. Introduction

The prevalence of myopia is rapidly increasing, with 50% of the population around the world estimated to be myopic and 10% to have high myopia in 2050 [1]. High myopes are at high risk of ocular complications, such as glaucoma, retinal detachment, and cataracts [2]. In the clinic, several optical interventions based on the peripheral myopic defocus mechanism have been prescribed for myopia control, such as orthokeratology, bifocal or multifocal soft contact lenses, and specialized spectacle lenses, based on evidence from some clinical trials [3–7]. Apart from the safety and efficacy of those interventions, it is also important to elucidate whether using them influences visual function.

Recent studies have shown that binocular vision changes after wearing orthokeratology lenses or multifocal soft contact lenses [8–10]. For example, the fusional range decreases [8], and ocular alignment shows an exophoric shift [8–10]. In these studies, subjects were always visually healthy with stable binocular vision; however, clinical concerns have been raised about whether those interventions can also be applied to children with abnormal binocular vision.

Intermittent exotropia (IXT) is the most common type of strabismus, seen in 3.24% of preschool children in China [11]. A population-based study showed that 46.5% of children with IXT were myopic by 10 years of age, and more than 90% were myopic by 20 years [12]. Considering the high prevalence of myopia, attention should be given to myopia progression in children with IXT. Given their differences from normal children, binocular vision is more critical for children with IXT, as if these individuals lose maintenance of fusion, they could manifest exotropia. Previous studies proved that the positive fusional vergence (PFV) and fusion reserve ratio were correlated with the ability to maintain ocular alignment [13–15], and PFV was lower in IXT than in visually normal children [16, 17], which could make it difficult for the fusional maintenance system to control ocular deviation. Since contact lenses for myopia control may influence the vergence and fusion systems in normal myopic children [8–10], therefore, it is worth exploring whether specialized spectacle lenses for myopia control will also affect binocular vision in myopic children, especially those with IXT.

Our recent study found that spectacle lenses with highly aspherical lenslets (HAL) effectively reduced the rate of myopia progression and axial elongation compared with single vision spectacle lenses (SVL) over two years [5]. In clinical practice, there may be some concerns about binocular vision when prescribing HAL for myopic children with IXT. Aiming to provide implications for prescribing HAL, we designed this study to explore the effect of HAL on binocular vision and accommodation for children with IXT and to explore the differences in the changes after wearing HAL in certain parameters between myopic children with or without IXT.

## 2. Methods

### 2.1. Subjects

Myopic children were recruited from the Eye Hospital of Wenzhou Medical University, China. Inclusion criteria included age between 9 and 12 years old, spherical equivalent ranging between -0.50 D and -6.00 D, astigmatism not more than 1.50 D, anisometropia less than 2.00 D, and monocular best spectacle-corrected visual acuity of 0.00 logMAR or better. The exclusion criteria were a history of ocular diseases other than IXT, ocular surgery, systemic diseases, and myopia control experience. Eligible subjects were enrolled into two groups based on phoria and the revised Newcastle Control Score (NCS) [18]. Subjects in the IXT group had exophoria of at least -10 prism diopters (PD) at a distance or near and an NCS of no more than 2 in each item. The visually normal group had orthophoria or exophoria no more than -10 PD at a distance or near. The study followed the tenets of the Declaration of Helsinki and was approved by the Ethics Committee of the Eye Hospital of Wenzhou Medical University, and informed consent was obtained from subjects and guardians.

**Figure 1:**
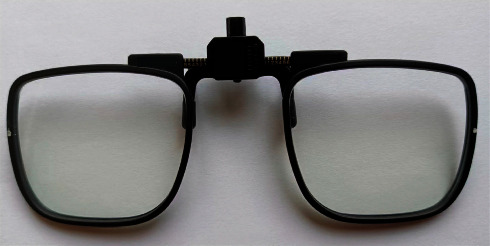
Testing lenses mounted into a particular trial frame whose bridge was adjustable.

During the experiment, the refractive error of each subject was fully corrected using single vision soft contact lenses (Biotrue, Bausch&Lomb Incorporated, NY, USA) with a special trial frame (Figure 1) for testing lenses. Two types of Plano testing lenses were added to both eyes in a random order: single vision spectacle lenses (SVL, Essilor Inc., Shanghai, China) and spectacle lenses with highly aspherical lenslets (HAL, Stellest, Essilor Inc., Shanghai, China) [6]. Both the testing lenses were made of polycarbonate.

### 2.2. Measurements

Subjects wore the assigned testing lenses for daily distance visual tasks for 20 minutes, such as looking at a distance, walking around but not allowed to read, or other near visual tasks. Then, the following measurements for binocular vision (details can be found in Appendix A in Supplementary Materials) were performed under 270 lux by an experienced optometrist who was blind to the lens type (lenslets were not obvious at the experimental luminance when face-to-face between the examiner and subjects): break point and spontaneous recovery point of fusional vergence, horizontal phoria at 3 m and 40 cm, near point of convergence (NPC), and vergence facility (VF). The convergence reserve ratio was calculated by dividing the convergence reserve by the angle of deviation [14].

The accommodative response (AR) was then measured by an open-field infrared autorefractor (WAM-5500, Grand Seiko Co. Ltd., Hiroshima, Japan). The subjects were asked to fixate on a rapid serial visual presentation of the text (story) at 33 cm under bilateral viewing conditions. Single black Chinese characters on a white background were presented sequentially on a computer screen and aligned with the right eye. The font size of the characters was 8-point size (angular subtense, 0.31°), and the rate of the presentation was 800 ms/character. The mean luminance of the target was 330 cd/m^2^. To obtain accommodative microfluctuation (AMF), the ARs were measured continuously for 60 seconds in a high-speed mode (5 Hz). AMF was defined as the standard deviation of AR [19]. Accommodative amplitude (AA), accommodative facility (AF), and stereoacuity were also evaluated following clinical procedures (details can be found in Appendix A in Supplementary Materials). AA and AF were measured for the right eye and both eyes.

The main outcome variables were fusional vergence, convergence reserve ratio, NPC, AA, AR, and AMF; the secondary variables included stereoacuity, phoria, AF, and VF. Examinations at a distance and ocular parameters related to relaxing eyes (such as negative fusional vergence) were performed first. Fusional vergence was repeated twice, phoria and AA were measured three times, NPC was repeated six times, and AF and VF were measured only once. The averages were used for analysis.

### 2.3. Data Analysis

The stereoacuities were converted to log values for analysis (from 1.30 to 2.90) [20]. If the stereoacuities were not measurable, the next log level above the largest disparity was assigned (3.20 for near stereoacuity and 2.90 for distance stereoacuity). Fusional vergence beyond 40 PD was arbitrarily recorded as 45 PD [14]. SPSS 26.0 was used for analysis. Normality was determined by the Shapiro–Wilk test and visual inspection of histograms. The *t*-test was used if data were normally distributed, and the Wilcoxon signed-ranks test was used for nonnormally distributed data. The differences in measured parameters between the two lenses were calculated, and absolute differences were calculated by subtracting the values of the SVL from those of the HAL. Relative differences were defined as the ratio of the absolute differences to the values of the SVL (absolute differences/values of SVL). If the values of the SVL between the two groups were comparable, the absolute differences were analyzed; otherwise, the relative differences were used. *P* < 0.05 was considered statistically significant.

## 3. Results

Forty subjects completed all measurements. The demographic and ocular characteristics of each group are shown in Table 1. No significant difference was found in age or refractive error between the two groups (all *P* > 0.05). Two subjects in the visually normal group and one in the IXT group were excluded from the analysis of AR and AMF because of discontinuous fixation. In four of the forty subjects, their test lenses were slightly tilted for capture measurements.

### 3.1. IXT Group

There were no significant differences in fusional vergence or convergence reserve ratio at a distance or near between HAL and SVL (all *P* > 0.05, Table 2). Compared with those after wearing SVL, AMF increased 0.04 D (95% CI, 0.03 to 0.05 D, *P*=0.001) after wearing HAL, while AR was not significantly influenced by HAL use (95% CI, −0.25 to 0.29 D, *P*=0.85, Table 2). NPC and AA showed no significant differences between the two lenses (all *P* > 0.05, Table 2). Comparisons in other parameters showed no significant differences (Appendix B1 in Supplementary Materials).

### 3.2. Visually Normal Group

No significant difference was found in fusional vergence between HAL and SVL (all *P* > 0.05, Table 3). AMF increased 0.05 D (95% CI, 0.03 to 0.07 D, *P*=0.001) after wearing HAL compared with SVL, and AR increased by 0.20 D (95% CI, −0.07 to 0.47 D, *P*=0.14). NPC and AA showed no significant differences between the two lenses (all *P* > 0.05, Table 3). No significant differences were found among the other parameters (Appendix B2 in Supplementary Materials).

### 3.3. Differences between the Two Lenses in the IXT and Visually Normal Groups

Fusional vergence was compared by ratio values, as the baseline values (with SVL) were significantly different between the IXT and visually normal groups. No significant differences were found for fusional vergence (all *P* > 0.05, Table 4). AR, AMF, NPC, and AA were not significantly different (all *P* > 0.05, Table 5). There were no significant differences among the other parameters (AppendixB3 in Supplementary Materials).

## 4. Discussion

Recently, HAL has been introduced in the clinic for myopia control, especially in China, but the effect on binocular vision remains unclear. This study assessed the influence of HAL on binocular vision and accommodation during initial wearing, especially for IXT children. Our results revealed that HAL did not significantly affect binocular vision and accommodation except for AMF; in addition, the changes after wearing HAL for the IXT group were not significantly different from those for the visually normal children.

AMF was increased in both groups after wearing HAL. AMF reflects the variability of accommodation, and its increase was possibly induced by variational accommodative stimuli from peripheral retina responses to the volume of peripheral myopic defocus, which was caused by aspherical HAL lenslets [6, 21]. Another possible factor influencing the greater AMF was the circumstances of initial wearing since the subjects in this study only adapted to the lenses for 20 minutes.

A previous study found that visual quality was decreased after wearing HAL in myopic children due to peripheral myopic defocus induced by aspherical lenslets [22]. Jainta et al. found that blurred images induced by simulated defocus (+0.50 D) could deteriorate convergence [23]. However, parameters of binocular vision (such as fusional vergence, NPC, and AA) were not significantly affected by HAL in this study. One main possible reason is that the loss in visual acuity caused by the lenslets was minimal, about half a line on a typical visual acuity chart (approximately 0.07 ± 0.09 logMAR) [22]. In addition, the subjects from Jainta's study were emmetropic adults [23]. A recent study demonstrated that children showed less sensitivity than adults to the blur induced by lenslets [24]; and myopes showed better blur adaptation than emmetropes [25, 26].

HAL did not significantly affect stereoacuity, phoria, AR, AF, or VF in this study. This may be because these measurements were evaluated when the subjects fixated on targets from the central area of the spectacles without lenslets. The differences between the two lenses in the IXT and visually normal groups were not significant; the possible reason was that subjects in the IXT group maintained fusion well during the study and to a degree similar to that of the visually normal subjects.

Binocular vision and accommodation were measured for the initial wearing of HAL in this study. Previous evidence showed that initial wearing was the most sensitive condition for displaying the effect on the visual system [8, 27–30]. First, human neuroadaptation can compensate for optical alterations with time goes [27–30]. In addition, previous studies, which investigated lenses designed with peripheral myopic defocus, have evaluated binocular vision and accommodation at the baseline and found that fusional vergence [31], phoria [31–34], NPC [32], AA [31, 35], AR [31, 35, 36], and AF [33, 35, 36] did not change with time. Based on these evidences, the long-term influence may keep consistent with that of short term if extending boldly. Thus, the findings of short-term influences in this study may have important implications for understanding the prescribing of HAL to visually normal children in clinical practice. However, the 20 minutes for adapting to the lenses might be too short for IXT subjects to manifest exotropia. In this study, the median NCS of the subjects with IXT was 1.0, which meant they had good fusional maintenance systems. The outcomes of the IXT group merely indicated the short-term influences on IXT children who can maintain fusion well. It remains to be determined whether the results can be extended to long-term effects for moderate or severe IXT children. Therefore, one limitation is that IXT children with deteriorated fusion were not included because they are generally treated with vision therapy or surgery in the clinic. Another limitation is that combination correction was used in this study. Refractive error was corrected using soft contact lenses, and two types of Plano testing lenses were induced randomly to compare binocular vision and accommodation function. Subjects were asked to wear lenses for 20 minutes of adaptation, and no subjects complained of discomfort with both contact lenses and trial spectacle lenses. Although the combination of contact lenses and spectacles is not commonly used in clinical practice, the comparison results of the two types of spectacle lenses were supposed not affected by the combination correction. Moreover, it is necessary to design a longitudinal study to explore the long-term influences of HAL use on different degrees of IXT.

## 5. Conclusions

For both mild IXT and visually normal children, HAL did not significantly influence binocular vision and accommodation except for AMF. AMF was increased in both groups, but it was not related to the presence or absence of IXT. Further studies are required to determine the long-term influences of HAL use on different degrees of IXT.

## Figures and Tables

**Table 1:** Demographic and ocular characteristics of the enrolled subjects.

	Visually normal group	IXT group	*T* Value	*P* Value
Age (years)	11.2 (1.2)	10.8 (0.9)	1.34	0.19

Sex (male: female)	11 : 9	11 : 9	0.01	0.99

OD SER (D)	−2.03 (0.83)	−1.99 (1.14)	−0.12	0.91

OS SER (D)	−2.25 (0.92)	−1.93 (1.26)	−0.91	0.37

OD astigmatism (D)	−0.45 (0.30)	−0.33 (0.33)	−1.11	0.28

OS astigmatism (D)	−0.45 (0.54)	−0.26 (0.35)	−1.35	0.19

OD residual SER (D)	0.04 (0.25)	−0.01 (0.19)	0.79	0.43

OS residual SER (D)	0.09 (0.21)	0.03 (0.27)	0.74	0.47

OD residual astigmatism (D)	−0.41 (0.28)	−0.48 (0.29)	0.69	0.50

OS residual astigmatism (D)	−0.55 (0.34)	−0.51 (0.33)	−0.35	0.73

Phoria at a distance (PD)	−0.6 (1.4)	−14.6 (6.2)	9.78	0.001

Phoria at near (PD)	−1.0 (1.8)	−20.0 (9.6)	8.70	0.001

Revised Newcastle control score^†^		1.0 (3.0)		

Data are presented as means (SDs), and ^†^data are presented as the median (IQR). The residual refractive error was determined using over-refraction. Abbreviations: IXT, intermittent exotropia; SER, spherical equivalent refractive error; D, diopters; PD, prism diopters.

**Table 2:** Binocular vision and accommodative parameters with HAL or SVL in the IXT group.

	HAL	SVL	Difference (95% CI)	*T* Or *z* value	*P* Value
Distance					

NFV break (PD)	13.4 (5.2)	13.6 (5.5)	−0.2 (−1.3, 0.9)	−0.38	0.71

NFV recovery (PD)	12.2 (3.4)	11.5 (3.6)	0.6 (−0.3, 1.6)	1.38	0.19

PFV break (PD)	16.3 (11.4)	15.6 (10.7)	0.7 (−0.9, 2.2)	0.92	0.37

PFV recovery (PD)	16.7 (9.7)	15.7 (9.7)	1.1 (−0.8, 3.0)	1.22	0.25

Convergence reserve ratio^†^	1.08 (0.58)	0.90 (1.43)		−0.28^‡^	0.78

Near					

NFV break (PD)	20.1 (8.2)	20.1 (8.8)	0.0 (−3.3, 3.3)	0.02	0.99

NFV recovery (PD)	16.4 (6.9)	17.2 (7.3)	−0.8 (−4.9, 3.3)	−0.41	0.69

PFV break (PD)	22.7 (13.4)	24.6 (14.2)	−1.9 (−5.0, 1.2)	−1.26	0.22

PFV recovery (PD)	20.2 (8.7)	20.3 (9.8)	0.0 (−2.4, 2.3)	−0.04	0.97

Convergence reserve ratio^†^	0.89 (1.29)	1.04 (1.33)		−1.44^‡^	0.15

NPC (cm)	5.19 (1.34)	5.11 (1.09)	0.08 (−0.16, 0.31)	0.67	0.51

Binocular AA (D)	15.42 (3.03)	16.06 (3.14)	−0.64 (−1.60, 0.31)	−1.41	0.17

Monocular AA (D)	13.21 (2.53)	13.59 (3.13)	−0.38 (−1.04, 0.28)	−1.19	0.25

AR (D)	1.69 (0.30)	1.67 (0.40)	0.02 (−0.25, 0.29)	0.16	0.88

AMF (D)	0.27 (0.06)	0.23 (0.08)	0.04 (0.03, 0.05)	6.80	0.001

Data are presented as means (SDs), and ^†^data are presented as medians (IQRs), ^‡^*z* value. Abbreviations: IXT, intermittent exotropia; HAL, spectacle lenses designed with highly aspherical lenslets; SVL, single vision spectacle lenses; NFV, negative fusional vergence; PFV, positive fusional vergence; NPC, near point of convergence; AA, accommodative amplitude; AR, accommodative response; AMF, accommodative microfluctuation; D, diopters; PD, prism diopters.

**Table 3:** Binocular vision and accommodative parameters with HAL or SVL in the visually normal group.

	HAL	SVL	Difference (95% CI)	*T* value	*P* Value
Distance					

NFV break (PD)	8.0 (2.1)	8.5 (2.1)	−0.5 (−1.3, 0.4)	−1.06	0.30

NFV recovery (PD)	5.7 (2.2)	6.3 (2.2)	−0.6 (−1.4, 0.2)	−1.58	0.13

PFV break (PD)	23.4 (11.3)	24.0 (13.2)	−0.6 (−3.9, 2.7)	−0.38	0.71

PFV recovery (PD)	16.5 (7.1)	15.8 (8.5)	0.8 (−1.8, 3.3)	−0.61	0.55

Near					

NFV break (PD)	13.5 (4.4)	13.4 (3.8)	0.1 (−1.6, 1.7)	0.06	0.95

NFV recovery (PD)	9.4 (1.0)	9.2 (4.6)	0.3 (−1.4, 1.9)	0.31	0.76

PFV break (PD)	39.2 (8.0)	40.5 (7.1)	−1.4 (−3.8, 1.1)	−1.17	0.26

PFV recovery (PD)	25.9 (6.5)	27.2 (8.0)	−1.4 (−7.6, 4.9)	−0.53	0.61

NPC (cm)	4.50 (0.52)	4.50 (0.75)	−0.11 (−0.32, 0.10)	−1.09	0.29

Binocular AA (D)	14.85 (2.93)	14.96 (2.29)	−0.11 (−1.07, 0.85)	−0.24	0.82

Monocular AA (D)	12.95 (2.37)	13.14 (2.15)	−0.19 (−0.77, 0.40)	−0.67	0.51

AR (D)	1.84 (0.44)	1.64 (0.34)	0.20 (−0.07, 0.47)	1.54	0.14

AMF	0.27 (0.06)	0.23 (0.08)	0.05 (0.03, 0.07)	6.10	0.001

Data were presented as means (SDs). Abbreviations: HAL, spectacle lenses designed with highly aspherical lenslets; SVL, single vision spectacle lenses; NFV, negative fusional vergence; PFV, positive fusional vergence; NPC, near point of convergence; AA, accommodative amplitude; AR, accommodative response; AMF, accommodative microfluctuation; D, diopters; PD, prism diopters.

**Table 4:** Relative differences in fusional vergence between the two lenses in IXT and visually normal groups.

	IXT group	Visually normal group	Difference (95% CI)	*T* value	*P* value
Distance					

NFV break (PD)	−0.2 (2.4)	−0.5 (1.9)	0.02 (−0.12, 0.16)	0.31	0.76

NFV recovery (PD)	0.6 (1.9)	−0.6 (1.7)	0.15 (−0.02, 0.33)	1.76	0.09

PFV break (PD)	0.7 (3.3)	−0.6 (7.1)	0.03 (−0.12, 0.19)	0.47	0.64

PFV recovery (PD)	1.1 (3.2)	0.8 (5.2)	0.07 (−0.17, 0.30)	0.59	0.56

Near					

NFV break (PD)	0.0 (7.1)	0.1 (3.5)	0.06 (−0.15, 0.25)	0.56	0.58

NFV recovery (PD)	−0.8 (7.9)	0.3 (3.6)	0.04 (−0.30, 0.38)	0.24	0.81

PFV break (PD)	−1.9 (6.7)	−1.9 (6.7)	0.00 (−0.14, 0.14)	0.01	0.99

PFV recovery (PD)	0.0 (3.7)	−1.3 (6.3)	0.12 (−0.14, 0.38)	0.98	0.34

Data are presented as means (SDs). Absolute differences are defined as HAL—SVL; relative differences are defined as absolute differences/values of SVL. Abbreviations: IXT, intermittent exotropia; HAL, spectacle lenses designed with highly aspherical lenslets; SVL, single vision spectacle lenses; NFV, negative fusional vergence; PFV, positive fusional vergence; PD, prism diopters.

**Table 5:** Absolute differences in visual parameters between two lenses in IXT and visually normal groups.

	IXT group	Visually normal group	Difference (95% CI)	*T* value	*P* Value
NPC (cm)	0.08 (0.50)	−0.11 (0.45)	0.18 (−0.12, 0.49)	1.22	0.23
Binocular AA (D)	−0.64 (2.04)	−0.11 (2.06)	−0.54 (−1.85, 0.78)	−0.83	0.41
Monocular AA (D)	−0.38 (1.41)	−0.19 (1.25)	−0.19 (−1.04, 0.66)	−0.45	0.66
AR (D)	0.02 (0.55)	0.20 (0.54)	−0.18 (−0.54, 0.19)	−0.98	0.33
AMF (D)	0.04 (0.02)	0.05 (0.04)	−0.01 (−0.03, 0.01)	−1.25	0.22

Data are presented as means (SDs). Absolute differences are defined as HAL—SVL. Abbreviations: IXT, intermittent exotropia; HAL, spectacle lenses designed with highly aspherical lenslets; SVL, single vision spectacle lenses; NPC, near point of convergence; AA, accommodative amplitude; AR, accommodative response; AMF, accommodative microfluctuation; D, diopters; PD, prism diopters.

## Data Availability

The data presented in this study are available on request from the corresponding author.

## Additional Points

The study was conducted in accordance with the Declaration of Helsinki and approved by the Ethics Committee of the Eye Hospital of Wenzhou Medical University (approval number: 2021-171-K-148-01) in 2021.

## Supplementary Materials

Supplementary Materials The details of methods in binocular vision and accommodation were showed in Appendix A. The comparisons of stereoacuity, phoria, accommodative facility, and vergence facility can be found in Appendix B.
