# Supplementary material for: Effect of Spectacle Lenses with Highly Aspherical Lenslets on Binocular Vision and Accommodation in Myopic Children with and without Intermittent Exotropia
Source: J Ophthalmol. 2022 Oct 12;2022:9306848. doi: 10.1155/2022/9306848 (PMC9581704; doi:10.1155/2022/9306848)
Supplement: Supplementary Materials — The details of methods in binocular vision and accommodation were showed in Appendix A. The comparisons of stereoacuity, phoria, accommodative facility, and vergence facility can be found in Appendix B. [file 9306848.f1.docx]

**Title:** Effect of spectacle lenses with highly aspherical lenslets on binocular vision and accommodation in myopic children with and without intermittent exotropia

Fengchao Zhou, BSc^12^, Xue Li, PhD^12^, Yingying Huang, MD^12^, Yuhao Li, BSc^12^, Jiali Zhang, BSc^12^, Hao Chen, MD, OD^12^, Jinhua Bao, PhD^12^

1. Eye Hospital and School of Ophthalmology and Optometry, Wenzhou Medical University, Wenzhou, Zhejiang, China

2. National Clinical Research Center for Ocular Diseases, Wenzhou, Zhejiang, China

Corresponding author:

Jinhua Bao, PhD, baojessie@163.com

Hao Chen, MD, OD, chenhao@mail.eye.ac.cn

Mailing address:

Eye Hospital of Wenzhou Medical University, 270 West Xueyuan Road, Wenzhou, Zhejiang, China, 325027

Tel: 86-0577-88068166

**Appendix A**

Fusional vergence was assessed using a 2 to 40 PD prism bar. A demonstration was performed prior to the measurements to allow the subjects to experience diplopia by placing a 20 PD BI prism in front of the right eye in children with visually normal children and a 40 PD BI prism for IXT children. The break point was determined by subjects reporting double images with no subsequent recovery. Spontaneous recovery was allowed for a short time after diplopia occurred. The recovery point was recorded when the subjects first fused double images to single images. If diplopia was not reported but the examiner noted exodeviation, the strength of the prism at which exodeviation occurred was recorded as the break point; under this condition, the recovery point was unable to be determined. Phoria was assessed using prism and alternate cover test with fixating a 0.2 logMAR target at distance (3 meters) and then at near (0.4 meters). The first prism neutralization endpoint was recorded. The near point of convergence was measured by the push-up method using the Royal Air Force near point rule (Bernell Corporation, Mishawaka, IN) with an accuracy of half a centimeter. During the measurement, the target was moved toward the subject, and the subject’s eyes were observed until the subject reported that the target appeared double or the examiner noted exodeviation. Record the distance as the break point. Vergence facility was measured at 40 cm using a 3BI/12BO vergence facility prism (Bernell Corporation, Mishawaka, IN) and a 0.2 logMAR target. A 30-second pilot exercise was practiced before formal measurements. Subjects were asked to try to fuse the targets and report “single” simultaneously; the examiner alternatively changed the strength of the prism as soon as possible after the subjects’ reports. Cycles were counted in one minute.

Accommodative amplitude was assessed similarly to near point of convergence. Subjects were instructed to keep the letters in N5 clear and report the first sustained blur. The accommodation facility was measured at 3 m and 40 cm using a plano/-2.00 D flipper and ±2.00 DS flipper, respectively, and 0.2 logMAR letters (all from Tianjin OPT Co. Ltd., Tianjin, China). A 30-second practice session was also performed prior. The subjects were asked to read the letters out as soon as the letters appeared clear. Cycles were counted in one minute. Stereoacuity was evaluated at 3 m and 40 cm by a Randot Stereotest. (Stereo Optical Company Inc, Chicago, IL).

**Appendix B**

**Appendix B1.** Comparisons of other parameters for the IXT group

|  | HAL | SVL | Difference (95% CI) | *T* value | *P* value |
| --- | --- | --- | --- | --- | --- |
| Distance |  |  |  |  |  |
| Stereoacuity | 2.29 (0.43) | 2.25(0.42) | 0.04 (-0.02, 0.09) | 1.28 | 0.22 |
| Phoria (PD) | -14.8 (5.5) | -15.3 (6.4) | 0.4 (-0.4, 1.3) | -1.03 | 0.31 |
| Binocular DAF (cpm) | 14.65 (3.66) | 14.02 (3.83) | 0.63 (-0.38, 1.63) | 1.30 | 0.21 |
| Monocular DAF (cpm) | 14.43 (3.50) | 14.63 (4.31) | -0.20 (-1.17, 0.77) | -0.43 | 0.67 |
| Near |  |  |  |  |  |
| Stereoacuity | 1.80 (0.38) | 1.79 (0.38) | 0.00 (-0.03, 0.04) | 0.23 | 0.82 |
| Phoria (PD) | -20.7 (9.5) | -20.5 (9.8) | -0.2 (-1.6, 1.1) | -0.33 | 0.74 |
| Binocular NAF (cpm) | 6.35 (3.19) | 6.65 (2.27) | -0.30 (-1.37, 0.77) | -0.59 | 0.56 |
| Monocular NAF (cpm) | 6.70 (3.10) | 6.78 (2.87) | -0.08 (-0.84, 0.69) | -0.21 | 0.84 |
| Vergence facility (cpm) | 11.66 (3.86) | 10.91 (4.78) | 0.75 (-0.73, 2.23) | 1.08 | 0.30 |

Data are presented as the means (SDs).

Abbreviations: DAF, accommodative facility at distance; NAF, accommodative facility at near; cpm, cycle per minute.

**Appendix B2.** Comparisons of other parameters for the visually normal group

|  | HAL | SVL | Difference (95% CI) | *T* value | *P* value |
| --- | --- | --- | --- | --- | --- |
| Distance |  |  |  |  |  |
| Stereoacuity | 2.19 (0.36) | 2.15 (0.37) | 0.04 (-0.02, 0.10) | 1.41 | 0.18 |
| Phoria (PD) | -0.7 (1.5) | -0.8 (1.5) | 0.2 (-0.1, 0.4) | 1.31 | 0.20 |
| Binocular DAF (cpm) | 14.13 (3.54) | 13.80 (4.34) | 0.33 (-0.99, 1.64) | 0.52 | 0.61 |
| Monocular DAF (cpm) | 14.93 (3.81) | 15.45 (3.94) | -0.53 (-2.02, 0.97) | -0.74 | 0.47 |
| Near |  |  |  |  |  |
| Stereoacuity | 1.71 (0.27) | 1.66 (0.28) | 0.05 (-0.01, 0.10) | 1.71 | 0.10 |
| Phoria (PD) | -0.5 (2.7) | -0.9 (2.3) | 0.4 (-0.5, 1.4) | 0.96 | 0.35 |
| Binocular NAF (cpm) | 6.43 (2.73) | 6.40 (2.28) | 0.03 (-0.84, 0.89) | 0.06 | 0.95 |
| Monocular NAF (cpm) | 6.60 (2.89) | 7.15 (2.76) | -0.55 (-1.52, 0.42) | -1.18 | 0.25 |
| Vergence facility (cpm) | 11.83 (5.23) | 11.88 (5.46) | -0.05 (-0.80, 0.70) | -0.14 | 0.89 |

Data are presented as the means (SDs).

Abbreviations: HAL, lenses designed with highly aspherical lenslets; SVL, single vision lenses; DAF, accommodative facility at distance; NAF, accommodative facility at near; cpm, cycle per minute; PD, prism diopters; D, diopters.

**Appendix B3.** Comparisons of other parameters between the IXT and visually normal groups

|  | IXT  Group | Visually  Normal group | Difference (95% CI) | *T* value | *P* value |
| --- | --- | --- | --- | --- | --- |
| Distance |  |  |  |  |  |
| Stereoacuity | 0.04 (0.12) | 0.04 (0.13) | -0.01 (-0.09, 0.08) | -0.13 | 0.90 |
| Phoria | 0.02 (0.18) | -0.39 (0.49) | 0.13 (-0.03, 0.30) | 1.98 | 0.10 |
| Binocular DAF (cpm) | 0.63 (2.16) | 0.33 (2.81) | 0.30 (-1.30, 1.90) | 0.38 | 0.71 |
| Monocular DAF (cpm) | -0.20 (2.08) | -0.53 (3.18) | 0.33 (-1.40,2.05) | 0.38 | 0.71 |
| Near |  |  |  |  |  |
| Stereoacuity | 0.00 (0.17) | 0.05 (0.12) | -0.04 (-0.11, 0.02) | -1.33 | 0.19 |
| Phoria | 0.04 (0.17) | -0.27 (0.75) | 0.14 (-0.08, 0.37) | 1.15 | 0.29 |
| Binocular NAF (cpm) | -0.30 (2.28) | 0.03 (1.84) | -0.33 (-1.65, 1.00) | -0.50 | 0.62 |
| Monocular NAF (cpm) | -0.08 (1.63) | -0.55 (2.08) | 0.48 (-0.72, 1.67) | 0.80 | 0.43 |
| Vergence facility (cpm) | 0.75 (2.78) | -0.05 (1.60) | 0.72 (-0.70, 2.13) | 1.03 | 0.31 |

Data are presented as the means (SDs). Phoria was compared with relative differences and other parameters were compared with absolute differences.

Abbreviations: IXT, intermittent exotropia; HAL, lenses designed with highly aspherical lenslets; SVL, single vision lenses; DAF, accommodative facility at distance; NAF, accommodative facility at near; cpm, cycle per minute; PD, prism diopters; D, diopters.
